# Is It Rape or Consent? College Men Just Don’t Know

**DOI:** 10.3390/ijerph23010038

**Published:** 2025-12-27

**Authors:** Stephanie A. Navarro Silvera, Eva S. Goldfarb, Amanda S. Birnbaum, Lisa D. Lieberman

**Affiliations:** Department of Public Health, Montclair State University, Montclair, NJ 07043, USA; goldfarbe@montclair.edu (E.S.G.); birnbauma@montclair.edu (A.S.B.); liebermanl@montclair.edu (L.D.L.)

**Keywords:** consent, sexual violence and misconduct, university campus, gender/sexuality, consent education and sex education

## Abstract

**Highlights:**

**Public health relevance—How does this work relate to a public health issue?**

- It has been recognized that sexual violence and misconduct present a major public health crisis on college campuses. Data indicate that women and LGBTQ+ undergraduates are at greater risk for sexual assault and rape than their cisgender male peers. This manuscript adds to literature that broadens the focus of campus sexual violence prevention by examining underlying social and environmental factors that contribute to campus dynamics related to sexual assault and rape. The literature indicates that it is important to consider emerging patterns in the understanding of consent by gender and sexual orientation in colleges’ efforts to address campus safety. This study sought to assess understanding of sexual consent among college students overall, and particularly among those most likely to commit sexual assault, namely cisgender heterosexual men.

**Public health significance—Why is this work of significance to public health?**

- This study provides support for a large and growing body of evidence suggesting that current practices about when, what, and for whom sexual assault prevention education is needed should be revisited.

**Public health implications—What are the key implications or messages for practitioners, policy makers and/or researchers in public health?**

- The results from this survey underscore the need for more comprehensive, gender-transformative education on consent. To reduce sexual assault, consent education including targeted strategies are necessary to engage cisgender heterosexual men in these discussions.

**Abstract:**

**Introduction:** Women and LGBTQ+ undergraduates face higher rates of sexual assault and rape compared to cisgender male peers—the overwhelming majority of perpetrators. Federal policies have aimed to curb campus sexual violence, yet questions remain about the efficacy of consent education, particularly among cisgender heterosexual men. **Methods:** This study surveyed 1567 undergraduate students at a large Northeastern public university in 2017 and 2022. Responses to a nine-item consent scale were analyzed by gender, sexuality, and demographic factors. **Results:** Cisgender heterosexual (cis-het) men had poorer understanding of consent compared to women and LGBTQ+ students, both in 2017 and 2022. Cis-het men’s consent scores showed no improvement, and for some items worsened from 2017 to 2022, while other groups showed significant improvements in recognizing the nuances of consent. **Conclusions:** Findings suggest that, despite increased focus on sexual assault prevention and social awareness campaigns like #MeToo, cisgender heterosexual men’s understanding of consent has not improved, highlighting the persistent challenge in shifting deeply ingrained beliefs about consent. Educational efforts should address these beliefs more directly, focusing on transforming societal norms around masculinity and sexual entitlement. **Policy Implications:** These results underscore the need for more comprehensive, gender-transformative education on consent. Current programs primarily focus on victims rather than perpetrators, which fails to address the root causes of sexual violence. To reduce sexual assault, consent education, delivered K-12 and in higher education, must consistently challenge harmful gender norms and promote equitable relationships. More targeted strategies are necessary to engage cisgender heterosexual men in these discussions.

## 1. Introduction

Women and LGBTQ+ undergraduates are at greater risk for sexual assault and rape than their cisgender male peers [1,2,3,4,5,6,7,8,9,10,11,12]. College women aged 18–24 are three times more likely than similarly aged non-student women, in general, to experience sexual violence [13], which affects one in four undergraduate females (26.4%) and nearly a quarter (23.1%) of transgender, genderqueer, and nonconforming (TGQN) people compared to 6.8% of undergraduate cisgender males [2]. Though minimal research explores why sexual assault and rape rates among Lesbian, Gay, Bisexual, Trans, and Queer/Questioning (LGBTQ) college students and women are so much higher than among heterosexual men, and while perpetration or victimization are not exclusive to any gender or sexual orientation, the majority of offenders in situations of rape or unwanted sexual contact, collectively referred to as sexual violence, continue to be men [14]. In fact, the 2006 National Violence Against Women Survey found that almost 86% of rape victims are female, nearly all of whom (99.6%) were raped by males, and that 85.2% of male victims were raped by a male [14]. A literature review conducted by Fedina et al. [5] further noted that approximately 19–25% of undergraduate female students reported some form of unwanted sexual contact compared to roughly 8% of male students.

### 1.1. Campus Efforts to Prevent Assault

College campuses are unique environments in which sexual encounters and relationships are facilitated by students’ being away from home, many for the first time, in large groups with others their age, so sexual violence and misconduct are also more prevalent [13]. Given that sexual violence and misconduct present a major health crisis on college campuses, in 2013, President Barack Obama signed into law *The Campus Sexual Violence Elimination (SaVE) Act*, as part of the *Violence Against Women Reauthorization Act* (42 USC §13,701). This bolstered *The Clery Act* of 1990, which requires federally funded colleges and universities to be transparent about crime on campus, provide support and accommodations to survivors of violence, and disclose efforts to improve safety on campus by, among other things, increasing reporting requirements by institutions of higher education related to sexual assault, dating, and domestic violence, and obligating them to provide campus-wide prevention and education programs [15]. These efforts have typically included education to decrease rape myth acceptance, namely, inaccurate or stereotyped beliefs about the causes and consequences of sexual violence [16], increase pro-social bystander behaviors such as intervening to prevent or halt a sexual assault [17]), raise awareness of sexual assault, decrease risky behaviors among women, and providing more robust support and reporting mechanisms for victims [18,19]. Research suggests that college efforts to address sexual assault and teach about consent can be effective in reducing rape culture beliefs on a personal and perceived campus level [20].

### 1.2. Understanding Consent

The definition of sexual consent (willingness to have sex) has continued to evolve from one based on not saying “no,” to affirmatively saying “yes” to participating in sexual activity [21], with affirmative consent being codified by the US Government in 2014 and becoming the standard among US colleges and universities shortly thereafter [22,23,24]. There is some evidence, however, that understanding of consent may vary by gender and sexuality [25,26,27].

Patterns in sexual assault perpetration and victimization by gender and sexual orientation, and emerging patterns in the understanding of consent by gender and sexual orientation, are important to consider in colleges’ efforts to address campus safety. The discussion of sexual assault was brought to the forefront of our collective conscience following the explosion of #MeToo in 2017 [28]. The wide and sustained recognition of the #MeToo movement between 2017 and 2022 provided the opening for a societal shift toward more open conversations about sexual violence and misconduct.

This study sought to assess understanding of sexual consent among college students overall, and particularly among those most likely to commit sexual assault, namely cisgender heterosexual men. Although it was not designed to assess the particular #MeTOO social movement, the timing of the two surveys has relevance in thinking about the potential impact of such movements.

## 2. Methods

### 2.1. Study Design and Sample

A survey of undergraduate students about their perceptions of the campus climate related to sexual violence and misconduct was first conducted in Spring 2017 at a large public university in a northeastern state using the online Survey Monkey platform. All undergraduate students with a valid university email were invited to complete the survey. The survey was distributed to all enrolled undergraduate students (~21,000) through their university emails. Reminders were sent two and four weeks after the initial invitation. Respondents had the option to enter into a drawing to win one of eight (8) gift cards of varying amounts (2- $75, 2- $50, 2- $25). The survey was repeated in 2022 using the Qualtrics system to which the institution had pivoted.

### 2.2. Study Measures

Understanding of consent was assessed using a nine-item consent scale developed and implemented by The Pennsylvania State University [29] with response options of agree, don’t know/not sure, or disagree (Appendix A). The same questionnaire was used at both T1 and T2. Gender was assessed by asking “Which best describes your gender?” with options including man, woman, non-binary/third gender, genderqueer, transman, transwoman, prefer not to say, and other; sexual orientation was assessed by asking “What is your primary sexual orientation?” with response options including lesbian, gay, bisexual, pansexual, and other. Gender categories were then recoded to man, woman, and trans+. The sexual orientation variable was recoded to heterosexual and LGB+.

After initial analyses, a combined variable was created to identify those who self-identified as both a cisgender man and heterosexual. Specifically, to create the gender/sexual orientation combined variable, participants who self-identified as both a cis-gender man (in the gender variable) and heterosexual (in the sexual orientation variable) were classified as cis-gender heterosexual man (i.e., “cis-het man”), and everyone who did not identify as a cis-gender heterosexual man were combined as “all others.”

Additional participant demographic information was gathered, including age, commuter status, student status (full- versus part-time), self-reported race/ethnicity, and participation in Greek life. Other survey items included questions regarding campus engagement, perceptions of the prevalence of sexual violence and misconduct on campus (harassment, assault, and rape), personal and friends’ experiences with campus sexual violence and misconduct, bystander experiences, perceptions about reporting and disclosure, depressive symptoms, and demographic characteristics [30]).

### 2.3. Data Analysis

A composite consent score was calculated by summing the responses to the nine consent assessment items (agree = 1, don’t know/not sure = 2, disagree = 3), with a higher score indicating a better understanding of consent (range 10–27). Item 7, “A person who says yes, or specifically states that they want to have sex with a partner, is consenting to sex,” was reverse-coded to indicate that agreement with this item is correct, contributing to a higher overall score. Reliability analysis of the scale yielded a Cronbach’s *α* = 0.77 at T1 and 0.68 at T2.

Differences in participant demographics by time of survey were assessed using chi-square analyses for variables including age, gender, sexual orientation, race, student status (commuter vs. residential student and full-time vs. part-time), and participation in Greek life. Bivariate analyses were then conducted using analysis of variance (ANOVA) to assess the association between potential explanatory variables and understanding of consent as measured using the calculated consent score, both overall and by year of survey. Next, differences for each individual consent item by year of survey, separately for cis-het men and all others, were conducted using chi-square analyses to identify changes over time for each subpopulation.

Finally, unconditional logistic regression was used to calculate an unstandardized beta score (β) and corresponding 95% confidence intervals (CIs) for the consent score and for each of the potential explanatory variables with mutual adjustment for variables included in the model. We utilized a backward Wald selection to obtain the most parsimonious model with both time of survey (2022 vs. 2017) and age (categorical) forced into the final models. Significance tests were two-sided, with a *p*-value of less than 0.05 considered statistically significant.

All analyses were performed using SPSS version 27.0 (IBM Corp., Released 2017. IBM SPSS Statistics for Macintosh, Version 27.0. Armonk, NY, USA: IBM Corp.).

## 3. Results

Survey respondents included 1353 students in 2017 and 992 in 2022. Of those, complete data were available for 868 students in 2017 and 758 students in 2022, totaling 1626 students who constituted the final sample. Demographic data overall, and for the two survey cohorts separately, are shown in Table 1. From the 2017 to the 2022 sample, there were some notable differences in respondents’ gender, with a statistically significant lower proportion of respondents identifying as men (22% and 15%, respectively, *p* < 0.001) and an increase in respondents identifying as trans+ (2% and 7%, *p* < 0.001), or women (75% and 78% *p* < 0.001). As well, there were statistically significant differences in respondents’ sexual orientation, with 85% of respondents identifying as heterosexual in 2017, and 63% in 2022 (*p* < 0.001). Further, between 2017 and 2022, the percentage of respondents who identified as cisgender heterosexual men fell from 19.8% to 12.6% (*p* < 0.001).

Table 2 shows differences in calculated consent scores by potential explanatory variables for the sample overall, as well as by year of survey. Calculated at each time frame separately, there were significant differences in consent scores by age in both 2017 and 2022. At both survey points, younger students’ higher scores reflected a more accurate understanding of consent than older students (*p* < 0.001 at T1, and *p* = 0.002 at T2). Students who lived on campus scored higher than commuters (*p* < 0.05 in both 2017 and 2022). As well, full-time student status yielded significantly better scores in 2017 (*p* < 0.001), and nearly significant (*p* = 0.06) in 2022. Racial/ethnic differences in consent scores were observed at both T1 and T2, with non-Hispanic White students scoring higher at both survey times, compared to all other racial/ethnic groups.

Notably, with respect to gender, men held significantly less informed views of sexual consent in both 2017 (*p* = 0.002) and 2022 (*p* < 0.001), compared to those identifying as either women or trans+. Regarding sexual orientation, those who identified as heterosexual had poorer (i.e., less informed) scores than LGB+ participants at both T1 (*p* < 0.001) and T2 (*p* < 0.001). The bivariate analysis of the consent scores by the combined gender/sexuality variable demonstrated that participants who identified as anything other than a cisgender heterosexual man had a more accurate understanding of sexual consent than cis-het men at both T1 (*p* < 0.001) and T2 (*p* < 0.001). The bivariate analysis of the consent scores by the combined gender/sexuality variable demonstrated that there were no significant differences between the women and LGBTQ+ groups. There were no significant differences on consent scores between women and LGBTQ+ respondents when analyzed separately. Subsequent analyses combined those two groups. For both groups (when analyzed separately and combined), those who identified as anything other than a cisgender heterosexual man had a more accurate understanding of sexual consent than cis-het men at both T1 (*p* < 0.001) and T2 (*p* < 0.001). Specifically, cisgender heterosexual men misinterpreted a wider range of situations as consensual than all other groups together.

The association between each consent item and the combined gender/sexual orientation variable for each year of the survey is provided in Table 3. Notably, while there were no statistically significant differences between 2017 and 2022 in the responses of cis-het men to 7 of the 9 consent items, there was statistically significant improvement in the scores among all others between T1 and T2 on 5 of the 9 consent items. Notably, cis-het men were more likely to agree with the statement “A person who looks like they want to have sex is consenting to sex” in 2022 (7.6%) than they were in 2017 (5.7%, *p* = 0.05) and were more likely to indicate that they did not know or were unsure if “A person who says yes, or specifically states that they want to have sex with the partner, is consenting to sex.” (2017 = 3.4%, 2022 = 10.8%, *p* = 0.04).

Given the likely multicollinearity of several of the explanatory variables, a multivariate linear regression was conducted, including characteristics that were significant at T1 and/or T2 of the bivariate analyses, using a stepwise selection for variable inclusion. The initial model included year of survey (2017 or 2022), age, commuter status, full/part-time student status, participation in Greek life, race, and the combined gender/sexual orientation variable. Using a stepwise selection, the model retained age, participation in Greek life, and gender/sexual orientation, with year of survey forced into the final model (Table 4). Notably, although there were differences in consent scale scores by race in the bivariate analyses, those differences were not significant in the final model. Likewise, the year of survey was not statistically significantly associated with composite consent score in the multivariate model, confirming, as the bivariate models suggested, that understanding of consent was not significantly different in 2022 from 2017 once gender/sexual orientation, age, and participation in Greek life were accounted for. As shown in Table 4, participants who were older, not affiliated with a Greek organization on campus, and identified as cis-het men had a poorer understanding of consent compared to those who were younger, Greek involved, and identified as anything other than a cis-het man.

## 4. Discussion

The data suggest that, overall, understanding of consent did not change much from 2017 (22.8 ± 3.7) to 2022 (22.6 ± 3.4), and that cis-het men’s scores declined, indicating a less accurate understanding of consent. The finding that understanding of what constitutes sexual consent was worse for cis-het men than all others at both T1 and T2, and that cis-het men’s scores on individual consent scale items remained the same or worsened in 2022, is a particular cause for concern, given that over 95% of American men identify as cisgender and heterosexual [31], and represent, by far, the largest number of perpetrators of rape, sexual assault, and harassment.

Current sexual assault prevention programs are largely geared toward those most likely to become victims, often focused on understanding the meaning of consent, preventing victimization, increasing bystander interventions, and making it easier for victims to report assaults to proper authorities [32]. A central criticism of these programs is that they do not rely on a public health approach, which focuses on mitigating the “upstream” underlying risk factors before they lead to sexually violent behavior [33,34], and are more focused on strengthening systems, structures, and social factors to eliminate sexual violence and misconduct [35].

### 4.1. Limitations

There are several notable limitations of this study. First, the study was undertaken at a single, albeit large, state university in a northeastern state. The sample itself was self-selected in that students chose to respond to the survey via a link in a university email (with several reminders). Although the sample may not be representative of the full campus community, the respondent group’s demographics (gender, race/ethnicity) were similar to, and reflected the shift in, the demographics of the university as a whole at both T1 and T2. Nonetheless, the change in the overall makeup of the student body could have affected perceptions of the campus environment. As well, the proportion of cisgender heterosexual men who participated in the survey was well below their representation in the population, and decreased between the first and second surveys. It may be that in 2022, more young adults overall self-identified as LGBTQ+ than in 2017 (as they did in this survey). As well, the increased attention to sexual assault and harassment in general, and on college campuses specifically, may have made cis-het men more fearful of responding. Whatever the reason, the relatively small number of cis-het men in our sample may have limited the robustness of our analysis and/or caused us to miss or to underestimate differences that only appear as non-significant trends in our analysis. It is important to note that sex and gender are different constructs. This survey did not ask about sex assigned at birth. The gender identity item, however, had categories for man, woman, transman, transwoman, genderqueer, and non-binary/third gender; thus we assumed that given these options, a transgender man would most likely have chosen the transgender category. It is possible that a few transgender men selected “man” instead of “transgender”, but that number is likely to have been very low in the context of the entire group who selected “man”. There is, in fact, no way to confirm this. The dramatic differences between this group and all others, however, suggest that the group of what we called “cisgender men” was predominantly, if not completely, men who were, in fact, cisgender. Future studies on this topic should include separate survey items for sex and gender. Even with these caveats, the findings are quite clear and compelling.

### 4.2. Implications and Future Research

This study provides support for a large and growing body of evidence suggesting that current practices about when, what, and for whom sexual assault prevention education is needed should be revisited. For example, extensive research suggests that introducing consent education in middle or high school grades is insufficient. As well, there is growing criticism of sexual violence and misconduct prevention efforts that focus on improving young people’s understanding of consent, arguing essentially that men do not rape because they do not know what consent is, but rather despite knowing what it is [36,37,38]. By and large, in our society, boys and men learn to integrate social norms about masculinity that encourage them to believe it is their responsibility to initiate sexual behaviors, and their right to engage in them. Leitch [39] argues that while the concept of consent is useful and effective in a legal context, it is inadequate in explaining our sexual obligations and values.

Research is needed to evaluate the effectiveness of educational approaches to reducing sexual violence on college campuses, and elsewhere, that are integrated into comprehensive sexuality education, that begin in early grades, and that include a focus on consent in the context of healthy relationships, bodily integrity, communication, boundaries, and agency [40,41,42].

## 5. Conclusions

The fact that those most likely to be perpetrators are less informed about sexual consent than those who are most likely to be victimized is disturbing, but not surprising, given the typical emphasis and approach of educational programming on this issue and the traditional sexual norms that exist. Why cisgender heterosexual men in this study appeared even less well-informed in 2022 than their counterparts in 2017, while all others seemed to have become more knowledgeable, is less clear, but a worrisome trend that needs further investigation.

Despite an increased focus on affirmative consent, and social trends such as the #MeToo movement bringing greater public awareness of the scope of the sexual assault problem, it is difficult to change deeply entrenched values and beliefs about sexual consent. Our data make clear that recent/current approaches are not working to change beliefs about sexual consent, particularly among cisgender heterosexual men. It is critically important that we launch new and different approaches to the work of sexual violence and misconduct prevention, including gender-transformative education, embedded within multi-faceted comprehensive sex education that begins in Kindergarten, and that focuses on preventing perpetration rather than victimization.

## Figures and Tables

**Table 1:** Participant demographics overall and by year of survey.

		Overall		2017		2022		*p*
		*N*	%	*N*	%	*N*	%	
Gender	Man	356	19.1	222	22.4	134	15.4	<0.001
	Woman	1431	76.8	750	75.5	681	78.3	
	Trans+	76	4.1	21	2.1	55	6.3	
Sexuality	Heterosexual	1375	75.2	845	85.4	530	63.2	<0.001
	LGB+	453	24.8	144	14.6	309	36.8	
Gender/ Sexuality	All others	1507	83.6	771	80.2	736	87.4	<0.001
	Cis-het men	296	16.4	190	19.8	106	12.6	
Age	18–19	678	34.2	298	30.0	380	38.3	<0.001
	20–21	703	35.4	389	39.2	314	31.7	
	22–24	407	20.5	240	24.2	167	16.8	
	25–30	119	6.0	45	4.5	74	7.5	
	>30	77	3.9	20	2.0	57	5.7	
Commuter	No	794	42.1	412	41.6	382	42.7	0.61
	Yes	1091	57.9	579	58.4	512	57.3	
Student status	Full-time	1748	92.7	932	93.9	816	91.4	0.04
	Part-time	138	7.3	61	6.1%	77	8.6	
Race	NH White	877	47.5	524	53.2	353	40.9	<0.001
	NH Black	241	13.0	102	10.4	139	16.1	
	Hispanic/Latine	546	29.5	264	26.8	282	32.7	
	NH AP/AI/NA	184	10.0	95	9.6	89	10.3	
Participation in Greek life	Never	1720	92.2	894	90.1	826	94.5	<0.001
	Previous or current	146	7.8	98	9.9	48	5.5	

**Table 2:** Consent score by potential explanatory variables, overall, and by year of survey.

Variable		Overall				2017				2022			
		N	Mean	SD	*p*	N	Mean	SD	*p*	N	Mean	SD	*p*
Gender	Man	307	21.6	4.1	<0.001	195	22.1	4.0	0.002	112	20.6	4.1	<0.001
	Woman	1238	22.8	3.4		653	23.0	3.6		585	22.5	3.2	
	Trans+	72	23.1	3.2		20	24.1	3.1		52	22.8	3.2	
Sexuality	Heterosexual	1195	22.4	3.7	<0.001	740	22.7	3.7	0.001	455	21.9	3.6	<0.001
	LGB+	392	23.2	3.1		125	23.8	3.2		267	23.0	3.0	
Gender/sexuality	All others	1310	22.8	3.4	<0.001	673	23.0	3.6	<0.001	637	22.5	3.2	<0.001
	Cis-het man	258	21.4	4.0		167	22.0	3.9		91	20.5	4.1	
Age	18–19	542	22.9	3.3	<0.001	266	23.4	3.4	0.005	276	22.5	3.1	0.002
	20–21	580	22.7	3.4		336	22.9	3.6		244	22.4	3.2	
	22–24	338	22.4	3.9		209	22.3	4.0		129	22.5	3.7	
	25–30	104	21.9	3.9		39	21.9	3.8		65	21.9	4.1	
	>30	61	20.6	4.1		17	21.3	3.5		44	20.3	4.3	
Commuter	No	683	23.1	3.3	<0.001	368	23.6	3.4	<0.001	315	22.5	3.2	0.10
	Yes	941	22.2	3.7		498	22.3	3.8		443	22.1	3.6	
Student status	Full-time	1508	22.7	3.5	<0.001	821	22.9	3.7	<0.001	687	22.3	3.3	0.08
	Part-time	118	21.3	3.8		47	21.0	3.8		71	21.6	3.9	
Race	NH White	780	23.1	3.2	<0.001	468	23.3	3.4	0.003	312	22.9	2.9	<0.001
	NH Black	198	22.3	3.6		85	22.3	4.0		113	22.2	3.3	
	Hispanic/Latinx	464	22.1	3.8		223	22.4	3.8		241	21.8	3.7	
	NH AP/AI/NA	165	21.6	4.0		86	22.2	4.2		79	21.0	3.8	
Participation in Greek life	Never	1499	22.5	3.6	0.008	782	22.7	3.7	0.03	717	22.2	3.4	0.04
	Previous or current	127	23.5	3.1		86	23.6	3.2		41	23.3	2.9	

**Table 3:** Bivariate analysis of individual consent items by gender/sex and year of survey.

	Cis-Het Men					All Others				
	2017		2022			2017		2022		
	*N*	%	*N*	%	*p*	*N*	%	*N*	%	*p*
A person who is engaging in foreplay is consenting to sex.					0.27					0.49
Agree	39	22.4	23	24.7		80	11.4	65	10.2	
Don’t know/not sure	29	16.7	22	23.7		69	9.8	74	11.6	
Disagree	106	60.9	48	51.6		552	78.7	501	78.3	
A person who removes their clothes or lets their partner remove their clothes is consenting to sex.					0.66					0.01
Agree	49	28.2	26	28.0		133	18.9	96	15.0	
Don’t know/not sure	28	16.1	19	20.4		56	6.6	67	10.5	
Disagree	97	55.7	48	51.6		523	74.5	478	74.6	
A person who does not push their partner away is consenting to sex.					0.59					0.02
Agree	22	12.6	13	14.1		81	11.6	46	7.2	
Don’t know/not sure	25	14.3	17	18.5		65	9.3	61	9.5	
Disagree	128	73.1	62	67.4		555	79.2	534	83.3	
A person who does not say “no” is consenting to sex.					0.38					0.005
Agree	21	12.3	11	11.8		85	12.1	44	6.9	
Don’t know/not sure	19	11.1	16	17.2		54	7.7	56	8.8	
Disagree	131	76.6	66	71		561	80.1	540	84.4	
A person who lets sexual activity progress to the point of intercourse is consenting to sex.					0.2					0.001
Agree	77	44	36	38.7		200	28.5	130	20.3	
Don’t know/not sure	23	13.1	20	21.5		92	13.1	106	16.5	
Disagree	75	42.9	37	39.8		409	58.3	405	63.2	
A person who looks like they want to have sex is consenting to sex.					0.05					0.88
Agree	10	5.7	7	7.6		16	2.3	14	2.2	
Don’t know/not sure	11	6.3	14	15.2		37	5.3	30	4.7	
Disagree	153	87.9	71	77.2		651	92.5	597	93.1	
A person who says yes, or specifically states that they want to have sex with the partner, is consenting to sex.					0.04					0.73
Agree	162	92.6	81	87.1		667	94.9	601	93.9	
Don’t know/not sure	6	3.4	10	10.8		15	2.1	17	2.7	
Disagree	7	4	2	2.2		21	3	22	3.4	
A person who talks about getting a condom is consenting to sex.					0.25					<0.001
Agree	42	24.3	25	26.9		200	28.4	129	20.2	
Don’t know/not sure	27	15.6	21	22.6		86	12.2	118	18.4	
Disagree	104	60.1	47	50.5		417	59.3	393	61.4	
A person who willingly goes somewhere private is consenting to sex.					0.82					0.81
Agree	10	5.7	7	7.5		28	4	28	4.4	
Don’t know/not sure	19	10.9	11	11.8		47	6.7	38	5.9	
Disagree	145	83.3	75	80.6		628	89.3	575	89.7	

**Table 4:** Multivariate linear regression* modeling potential explanatory variables and calculated consent score.

Variable		Unstandardized Beta	*p*	Lower	Upper
Year of response	2022 vs. 2017	0.3	0.09	−0.05	0.65
Age	18–19	-			
	20–21	−0.44	0.04	−0.85	−0.02
	22–24	−0.7	0.005	−1.18	−0.22
	25–30	−0.81	0.03	−1.55	−0.07
	>30	−1.88	<0.001	−2.82	−0.94
Greek life	yes/former vs. never	0.9	0.006	0.26	1.54
Gender/Sexuality	Cis-het man vs. all others	−1.33	<0.001	−1.80	−0.87

## Data Availability

The original contributions presented in this study are included in the article. Further inquiries can be directed to the corresponding author.

## Appendix A. Consent Questions

A person who is engaging in foreplay is consenting to sex.
A person who removes their clothes or lets their partner remove their clothes is consenting to sex.
A person who does not push their partner away is consenting to sex.
A person who does not say “no” is consenting to sex.
A person who lets sexual activity progress to the point of intercourse is consenting to sex.
A person who looks like they want to have sex is consenting to sex.
A person who says yes, or specifically states that they want to have sex with the partner, is consenting to sex.
A person who talks about getting a condom is consenting to sex.
A person who willingly goes somewhere private is consenting to sex.
